# Phenotypic variations in persistence and infectivity between and within environmentally transmitted pathogen populations impact population-level epidemic dynamics

**DOI:** 10.1186/s12879-019-4054-8

**Published:** 2019-05-22

**Authors:** Andrew F. Brouwer, Marisa C. Eisenberg, Nancy G. Love, Joseph N.S. Eisenberg

**Affiliations:** 1 Department of Epidemiology, University of Michigan, 1415 Washington Heights, Ann Abor, 48109 MI USA; 2 Department of Civil and Environmental Engineering, University of Michigan, 1351 Beal Avenue, Ann Arbor, 48109 MI USA

**Keywords:** Biphasic decay, Microbial dormancy, VBNC, Infectious disease transmission model, Identifiability, Persistence–infectivity trade-off

## Abstract

**Background:**

Human pathogens transmitted through environmental pathways are subject to stress and pressures outside of the host. These pressures may cause pathogen pathovars to diverge in their environmental persistence and their infectivity on an evolutionary time-scale. On a shorter time-scale, a single-genotype pathogen population may display wide variation in persistence times and exhibit biphasic decay.

**Methods:**

We use a transmission modeling framework to develop an infectious disease model with biphasic pathogen decay. We take a differential algebra approach to assessing model identifiability, calculate basic reproduction numbers by the next generation method, and use simulation to explore model dynamics.

**Results:**

For both long and short time-scales, we demonstrate that epidemic-potential-preserving trade-offs have implications for epidemic dynamics: less infectious, more persistent pathogens cause epidemics to progress more slowly than more infectious, less persistent (labile) pathogens, even when the overall risk is the same. Using identifiability analysis, we show that the usual disease surveillance data does not sufficiently inform these underlying pathogen population dynamics, even when combined with basic environmental monitoring data. However, risk could be indirectly ascertained by developing methods to separately monitor labile and persistent subpopulations. Alternatively, determining the relative infectivity of persistent pathogen subpopulations and the rates of phenotypic conversion will help ascertain how much disease risk is associated with the long tails of biphasic decay.

**Conclusion:**

A better understanding of persistence–infectivity trade-offs and associated dynamics can improve our ecological understanding of environmentally transmitted pathogens, as well as our risk assessment and disease control strategies.

**Electronic supplementary material:**

The online version of this article (10.1186/s12879-019-4054-8) contains supplementary material, which is available to authorized users.

## Background

Many human pathogens, particularly waterborne enteric pathogens, require a host to reproduce but are transmitted through the environment where they are subjected to a variety of stressors. These stressors can result in long-term selection pressure that causes pathogens to evolve over time. The most widely studied evolutionary trade-off is the transmission–virulence trade-off [1], although others, such as the virulence–persistence trade-off [2] or the invasion–persistence trade-off [3], have also been examined. Short-term environmental stressors (e.g., related to temperature, salinity, pH, nutrient load), on the other hand, can also lead to different dynamics in different environmental conditions. Changes in kinetics [4], morphology [5], or antimicrobial resistance [6] can occur in response to environmental changes on this time-scale. The trade-offs resulting from these various environmental pressures add complexity to infectious disease systems and are difficult to study and predict. Models are useful for elucidating how phenotypic heterogeneity between and within pathogen populations impacts disease system dynamics. These dynamical insights can in turn help to develop effective environmental monitoring plans and to optimize risk-reduction interventions.

The focus of this analysis is exploring the public health implications of phenotypic variation between and within environmentally transmitted pathogen populations and how different kinds of data—both experimental and epidemiological—will be needed to inform our models of the underlying systems. In particular, we are interested in the implications of variations in and potential trade-offs between *persistence* in the environment, i.e., how long the pathogen remains viable outside the host, and pathogen *infectivity*, i.e., the probability of host infection given exposure to the pathogen. Microbiological research in infectious disease systems has largely emphasized the identification of genes, gene expression, and metabolic pathways that are associated with virulence, but this work has not translated well into better understanding of risk and disease dynamics at the population level [7]. Although virulence is important to public health, it is infectivity that primarily drives transmission and is therefore integral to risk assessment and control.

Variations in persistence times and infectivity can be seen between closely related pathogen species or pathovars, such as is seen for *Escherichia* and *Salmonella* genera. *E. coli*, in particular, is an extraordinarily diverse group, with only about 6% of gene families represented in every genome [8]. *E. coli* includes several pathovars that can cause enteric disease, and, although *E. coli* pathovars differ in their infectivity and persistence [9], these differences are not well characterized as a whole. There are clues, however, as to how trade-offs at the genetic and metabolic level can propagate to persistence and infectivity phenotypes at the pathogen population level. Environmental persistence, for example, may be driven by genes coding for resistance to specific stressors (e.g., resistance to higher temperatures, differences in pH, or salinity) [10], for pathways to utilize alternate energy sources [11], or the ability to infect and survive in amoebas or other protozoa [12, 13]. Infectivity, on the other hand, may be dependent on whether the infection mechanism acts locally or systemically in the host [14, 15], as well as the effectiveness of the infection mechanism. While evolutionary trade-offs between environmental persistence and infectivity have been demonstrated theoretically [16], in practice they are likely moderated by trade-offs with other life-history components [17]. Ultimately, evolutionary pressure may direct genetic and metabolic trade-offs across pathovars, resulting in a spectrum of persistence–infectivity strategies with implications for human health.

**Fig. 1:**
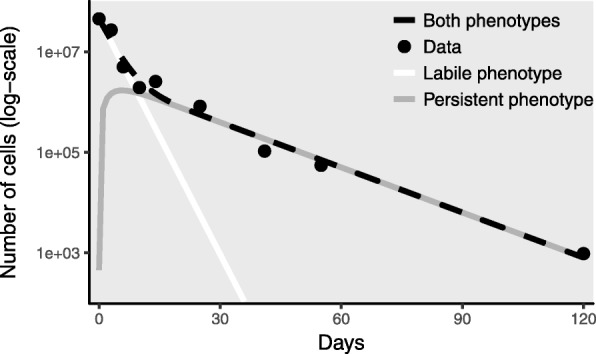
Phenotypic heterogeneity in pathogen persistence leads to biphasic decay. Biphasic decay of *E. coli* observed in manure-amended soil by [21] can be explained by a model in which fast-decaying labile pathogens transition to a slow-decaying persistent phenotype [18]. If the persistent phenotype represents a dormant state or has otherwise reduced infectivity, these underlying dynamics have important implications for host-level disease outcomes

Variation in persistence within a single-genotype population, on the other hand, can be observed as biphasic decay (Fig. 1), i.e., long-tailed deviations from the expected monophasic exponential pathogen decay [18]. Biphasic decay is well-documented in *E. coli*, for instance [19–23]. While the mechanisms of biphasic decay are not well-understood, hypotheses include genetic heterogeneity, hardening-off, and the existence of dormant states, such as viable-but-not-cultivable (VBNC) [24–26] or antibiotic-resistant persister [27–29] states. Whether prolonged persistence of pathogens presents a significant public health risk remains an open question. Many risk assessments do not account for this change in persistence. For example, published risk assessments of *Helicobacter pylori* have used an infectivity based on a less-persistent, culturable state [30], despite the fact that *H. pylori* transforms to a more-persistent but less-infectious VBNC state within days of entering water [5, 31]. In general, while it is likely that the persistent phenotype must sacrifice all or part of its infectivity (at least until it finds more favorable conditions), experimental verification is lacking for most pathogens.

Here we examine the dynamical properties associated with a trade-off between persistence and infectivity and the implications for future microbiological work and improved environmental monitoring strategies. We previously developed a mechanistic mathematical framework to describe biphasic decay, both in sampling studies and in quantitative microbial risk assessments [18]. We now extend that work by explicitly examining the dynamic implications at the host population level of a persistence–infectivity trade-off at the pathogen level. Specifically, we i) use an existing transmission model to consider the different outbreak dynamics that can be seen for pathogens across a variety of persistence–infectivity strategies and ii) present a new model to consider how within-population phenotypic heterogeneities can further affect outbreak dynamics.

These questions consider how underlying biological pathogen mechanisms and phenotypic variations translate into host-level dynamics, i.e., what patterns arise from the processes. The reverse question is also important, i.e., when can we infer the process from the pattern. Here, we want to know the extent to which we can make inferences about the persistence–infectivity trade-off from epidemiological time-series data. It is not clear a priori when longitudinal disease surveillance contains enough information to untangle more complex underlying mechanisms. The field of identifiability has developed methods to determine which model parameters can be uniquely estimated from a given kind of data. This information can then be used to ascertain which experiments or new data collection will have the most power for improving inference. We use identifiability analysis to highlight the ways in which targeted experimental studies could elucidate underlying mechanisms and improve our understanding of pathogen ecology and evolution, as well as risk assessment and disease control practices.

## Methods

### Models

**Fig. 2:**
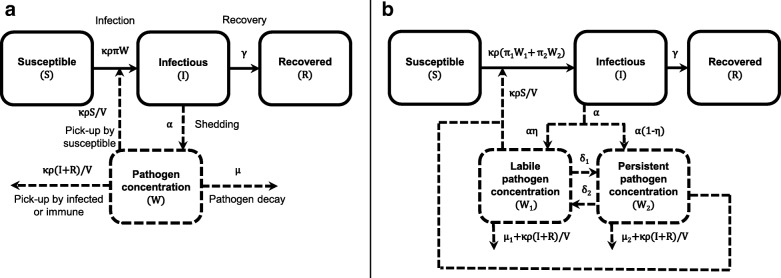
Environmentally mediated infectious disease transmission models. Schematics for models with **a**) monophasic pathogen decay and **b**) biphasic pathogen decay, where the pathogen population is comprised of a more infectious, less persistent labile fraction and a less infectious, more persistent fraction

We use an environmentally mediated infectious disease transmission model based on a susceptible–infectious–recovered (SIR) framework where all transmission occurs via an environmental compartment and there is no direct person-to-person transmission [32, 33]. This model incorporates an environmental compartment *W* that represents the concentration of pathogens in an environmental reservoir. Infectious people shed into this compartment (at rate *α*), and individuals contact the environment, picking up pathogens (with contact rate *κ* and per contact volume *ρ*). This model and variations of it have been used to explore the role of the environment in waterborne, airborne, and fomite-mediated transmission (e.g., [32–35], and many others [36]). In this first model, all pathogens in the population have the same *infectivity* (per-pathogen infection probability *π*). Previous work has shown that a linear dose–response function is sufficient to capture epidemic dynamics in most instances [37]. Moreover, all pathogens decay with the same monophasic exponential rate (*ξ*), leading to the same average *persistence*, that is, average number of days until removal from the system *τ*=1/*ξ*. Model variables and parameters are given in Table 1, and a schematic is given in Fig. 2a.

**Table 1:** Variables and parameters of the environmentally mediated infectious disease models

Variables	
*S*(*t*)	Number of susceptible people
*I*(*t*)	Number of infectious people
*R*(*t*)	Number of recovered people
*W*(*t*)	Concentration of pathogens in the environment
*W*_1_(*t*)	Concentration of labile pathogens in the environment
*W*_2_(*t*)	Concentration of persistent pathogens in the environment
Parameters	
*γ*	Recovery rate (per day)
*α*	Deposition rate of pathogens per unit volume of environment (per day)
*η*	Fraction of deposited pathogens that are labile
*δ*_*i*_	Rate at which pathogen of phenotype *i* convert to the other phenotype (per day)
*π*_*i*_	Per-pathogen probability of infection for phenotype *i*
*μ*_*i*_	Pathogen decay rate for phenotype *i* (per day)
*κ*	Rate at which individuals contact the environment (per day)
*N*	Population size
*ρ*	Volume of environment consumed (per contact)
*V*	Total volume of the environment
*ξ*_*i*_	Overall pathogen removal rate for phenotype *i* (per day), *μ*_*i*_+*κρN*/*V*
*τ*_*i*_	Average persistence of pathogen phenotype *i* (days)
*ϕ*_*i*_	Probability that conversion from phenotype *i* occurs before decay

Models incorporate monophasic pathogen decay (Eq. (1), Fig. 2a) or biphasic decay (Eq. (1), Fig. 2b)

$$ \begin{aligned} \dot S&= - \kappa \rho \pi S W, \\ \dot I&= \kappa \rho \pi S W-\gamma I,\\ \dot R& = \gamma I,\\ \dot W&= \alpha I - \xi W. \end{aligned}  $$

We extend the above model to account for biphasic decay [18] and allow for heterogeneities in population persistence and infectivity. In particular, we assume that the population consists of two phenotypes, one that is more infectious but less persistent (labile pathogens *W*_1_) and one that is less infectious but more persistent and may represent a dormant state (persistent pathogens *W*_2_). These subpopulations differ in phenotype (gene expression or metabolism) rather than genotype (DNA sequence). Model variables and parameters are given in Table 1, and a schematic is given in Fig. 2b. This model allows us to consider the state in which pathogens are shed into the environment (either *W*_1_ or *W*_2_, determined by *η*) and the possibility of phenotype conversion from labile to persistent and from persistent to labile (*δ*_1_ and *δ*_2_, respectively). One or more of these parameters might be negligibly small in practice (e.g., all pathogens are shed as the first phenotype (1−*η*=0), or persistent pathogens never regain their infectivity (*δ*_2_=0)), which would appreciably simplify the model and its identifiable parameter combinations.

1 $$\begin{array}{*{20}l} \dot S&= - \kappa\rho S(\pi_{1}W_{1}+\pi_{2}W_{2}),\\ \dot I&= \kappa \rho S(\pi_{1}W_{1}+\pi_{2}W_{2})-\gamma I,\\ \dot R &= \gamma I,\\ \dot W_{1}&= \alpha \eta I + \delta_{2} W_{2} - \left(\xi_{1} +\delta_{1}\right)W_{1},\\ \dot W_{2}&= \alpha (1-\eta) I + \delta_{1} W_{1} - \left(\xi_{2}+\delta_{2} \right)W_{2}. \end{array} $$

The two subpopulations can have different *infectivities* (*π*_1_ and *π*_2_). The average *persistence* *τ*_*i*_ for each pathogen type is the average amount of time a pathogen stays in a compartment and now includes both removal by decay or pick-up (*ξ*) and phenotypic conversion (*δ*): *τ*_*i*_=1/(*ξ*_*i*_+*δ*_*i*_). When the subpopulations have the same infectivity and removal rates (*π*_1_=*π*_2_,*ξ*_1_=*ξ*_2_), the model simplifies to the monophasic decay model above (Eq. (1)).

### Parameter identifiability: estimation and dynamic invariants

Direct measurement of the rates of biological processes or other mechanistic parameters through experimental studies is one way to learn about the underlying biological systems. However, sometimes experiments are inconvenient, expensive, or (especially in the case of pathogen challenge studies to determine infectivity) ethically fraught. Indirect methods—using the observation of the system dynamics to determine what the biological parameters must have been—have played an important part in infectious disease epidemiology in particular. However, we may be limited in what we can infer from time-series data, particularly when the underlying processes are complex.

Identifiability is the study of what model parametric information is available in data. A model parameter is *identifiable* if its value may be uniquely recovered from the observed data and not identifiable if multiple values could all lead to the same data. For example, one could never separately and uniquely estimate *m*_1_ and *m*_2_ in the linear model *y*=(*m*_1_+*m*_2_)*x*+*b*, no matter how many (*x*,*y*)-pair data points were measured. Unlike in this example, determination of these sorts of structural limitations is non-trivial for models of even modest complexity. For more formal definitions and discussions of identifiability, we refer the reader to the foundational work of [38, 39] and as well as several reviews [40, 41].

Identifiability analysis is a necessary precursor to parameter estimation because we cannot estimate the value of a parameter if multiple values all give the same output. If not all model parameters can be identified from data, one can find algebraic combinations of the parameters that are identifiable from the data [40]. In this example, *m*=*m*_1_+*m*_2_ can be uniquely estimated from the data. These *identifiable parameter combinations* are central to identifiability analysis and the model dynamics: any set of individual parameter values with the same value of their algebraic combination will produce the same dynamics. This means that the identifiable parameter combinations are invariants for the system dynamics.

Parameters that are indistinguishable when measuring one kind of data, however, might be separable for a different kind. Thus, identifiability analysis can also tell us how useful new experimental or observational studies would be to our inference by determining which model parameters or variables change the identifiability of our quantities of interest. In this example, having independent experimental determination of the value of *m*_1_ (allowing us to fix its value in the model) would render *m*_2_ identifiable.

To compute the identifiable parameter combinations, we use a differential algebra approach to identifiability, which is detailed elsewhere [42–44]. In brief, this method converts the system of equations into an input–output equation, which is a monic, polynomial equation that can be written in terms of only the observed state variable (i.e., the data variable), its derivatives, and the model parameters. The input–output equation has equivalent observed dynamics to that of the original ODE system, and the coefficients of the input–output equation are the identifiable parameter combinations. Mathematical details and proofs are left to the supplementary material.

Even if there is no theoretical structural barrier to estimating a parameter from data, there may be practical barriers present in real-world data sets, such as insufficient temporal resolution or the lack of time points around crucial features of the dynamic trajectory. Sometimes models are robust, producing almost indistinguishable behavior as a parameter changes several orders of magnitude. These real-world uncertainties are assessed by practical identifiability, which contrasts with the structural identifiability discussed above. In this analysis, we will primarily discuss structural identifiability, though we will touch on issues of practical identifiability in the simulation example.

### Basic reproduction number

The *basic reproduction number*$\mathcal {R}_{0}$, defined as the average number of secondary cases arising from a typical primary case in an entirely susceptible population, is often used for its epidemic threshold properties, i.e., for initial conditions near the disease-free equilibrium, there will be an epidemic if $\mathcal {R}_{0}>1$, and the disease will die out if $\mathcal {R}_{0}<1$. The basic reproduction number is also used to determine needed intervention coverage to eliminate transmission and to estimate the final size of an epidemic. Here, we will use $\mathcal {R}_{0}$ as a proxy for pathogen fitness, investigating different persistence and infectivity combinations that have the same $\mathcal {R}_{0}$. We calculate $\mathcal {R}_{0}$ for our ODE models using the Next Generation Method [45, 46]. For the models presented here, the basic reproduction number determines the epidemic attack ratio, i.e., the proportion of the at-risk population that develops the disease during the outbreak, also known as the cumulative incidence [33].

### Computation

Integration of ODE models was done in R (v3.4.1) with the deSolve package [47], and parameter estimation was done using a David–Fletcher–Powell algorithm in the Bhat package [48]. The differential algebra computation for the identifiability analysis was done in Mathematica (v11.1).

## Results

This first section presents the basic reproduction number and the identifiable parameter combinations for both the monophasic and biphasic pathogen decay models. We next elucidate what data are need to fully identify these environmentally mediated transmission models, and finally we examine the how persistence–infectivity trade-offs affect the transmission dynamics.

### The basic reproduction number and identifiability

#### Monophasic decay disease model

The basic reproduction number for the model with monophasic decay is given by [32, 33]

2 $$ \mathcal{R}_{0}= \frac{\alpha \pi\kappa\rho\tau N}{\gamma}.   $$

The identifiable combinations of this model have been published elsewhere [49]. In brief, if case data (corresponding to state *I*) are observed, then the observed dynamics are determined by the recovery rate *γ*, the average pathogen persistence *τ*, and the product *απκρ*. The basic reproduction number $\mathcal {R}_{0}$, therefore, is structurally identifiable if the population size *N* is known.

The parameter combination *απκρ* can be understood in the following way. The product *κρ* is the volume of the environment ingested per day, and *π* is the per pathogen probability of infection. Then, *πκρ* is the rate of infection-transmitting contact with the environment. The shedding rate (*α*) and infectious contact rate (*πκρ*) are in an identifiable parameter combination when we only observe infections in the population (*I*). In this case, we do not measure the concentration of pathogens in the environment (*W*), and we do not know whether the force of infection (*πκρW*) is a result of fewer pathogens that have a higher rate of infection (low shedding *α*, high rate of infectious contact *πκρ*) or more pathogens with a lower rate of infection (high shedding *α*, low rate of infectious contact *πκρ*). If the concentration of pathogens in the environment is observed, however, the relative sizes of the shedding rate (*α*) and infectious contact rate (*πκρ*) are distinguishable. That is, if environmental monitoring data (*W*) is also available in addition to case data (*I*), then *α* and *πκρ*, not just their product, are separately identifiable.

#### Biphasic decay disease model

In the biphasic pathogen decay model (Eq. (1)), pathogens leave their environmental compartment either by decay (*ξ*_*i*_) or by phenotype conversion (*δ*_*i*_), and we denote the probability that conversion occurs before decay by *ϕ*_*i*_:=*δ*_*i*_/(*ξ*_*i*_+*δ*_*i*_). It is easier to interpret the basic reproduction number $\mathcal {R}_{0}$ and certain identifiable parameter combinations of this model in terms of *ϕ* and *τ* rather than *ξ* and *δ*. The basic reproduction number is

3 $$ {\begin{aligned} \mathcal{R}_{0}&= \frac{\alpha\kappa\rho N}{\gamma}\left(\pi_{1}\tau_{1}\left(\frac{\eta+(1-\eta)\phi_{2}}{1-\phi_{1}\phi_{2}}\right)\right. \\& \quad \left.+ \pi_{2}\tau_{2}\left(\frac{(1-\eta)+\eta \phi_{1}}{1-\phi_{1}\phi_{2}}\right)\right).  \end{aligned}}  $$

The calculations are left to the Additional file 1. This system $\mathcal {R}_{0}$ can be seen as the sum of two submodel basic reproduction numbers that give the contributions of the labile ($\mathcal {R}_{0,1}$) and persistent ($\mathcal {R}_{0,2}$) phenotypes to the overall basic reproduction number.

4 $$ {\begin{aligned} \mathcal{R}_{0}&= \left(\frac{\alpha\pi_{1}\kappa\rho\tau_{1} N}{\gamma}\right) \left(\frac{\eta+(1-\eta)\phi_{2}}{1-\phi_{1}\phi_{2}}\right)\\&+\left(\frac{\alpha\pi_{2}\kappa\rho\tau_{2} N}{\gamma}\right)\left(\frac{(1-\eta)+\eta \phi_{1}}{1-\phi_{1}\phi_{2}}\right), \end{aligned}}  $$

5 $$ {\begin{aligned} \quad&\,=:\mathcal{R}_{0,1}+\mathcal{R}_{0,2}. \end{aligned}}  $$

These two submodels are each similar in form to the monophasic basic reproduction number (Eq. (2)), with a coefficient that accounts for the interconnectedness of the two compartments. Of the *α* pathogens shed between the two compartments, *αη* go directly to the labile compartment, but *α*(1−*η*)*ϕ*_2_ will also come to the labile compartment via the persistent compartment. These two pathogen sources explain the numerator of the interconnectedness coefficient, i.e., *η*+(1−*η*)*ϕ*_2_. Next, because pathogens can move back and forth between compartments, we need to know the expected number of visits a pathogen makes to the labile compartment [46]. After the initial visit, each return visit happens with probability *ϕ*_1_*ϕ*_2_. Thus, the expected amount of time spent in the labile compartment is

6 $$ \tau_{1}(1+\phi_{1}\phi_{2}+(\phi_{1}\phi_{2})^{2}+\cdots+(\phi_{1}\phi_{2})^{n}+\cdots)= \frac{\tau_{1}}{1-\phi_{1}\phi_{2}}.  $$

This term explains the denominator of the interconnectedness coefficient.

Because these submodel reproduction numbers allow us to understand the relative contribution of each pathogen phenotype to the overall epidemic potential of the system, we would like to be able to determine their values from time-series data. In particular, we want to understand the risk potential in the less infectious, persistent fraction of pathogens. However, it is not clear a priori whether we can determine these risk potentials from time-series data alone, and so we need identifiability analysis to determine the identifiable parameter combinations for the biphasic decay model and to inform what data are required to provide useful information from the model. Mathematical computation and details are left to the Additional files 1, 2, 3, 4 and 5.

If we only have human disease surveillance time-series (case data, *I*), then the observed dynamics are determined by

$$\gamma,$$

$$\alpha(\eta\pi_{1}+(1-\eta)\pi_{2})\kappa\rho,$$

$$\xi_{1}+\delta_{1}+\xi_{2}+\delta_{2}=\frac{\tau_{1}+\tau_{2}}{\tau_{1}\tau_{2}},$$

$$(\xi_{1}+\delta_{1})(\xi_{2}+\delta_{2})-\delta_{1}\delta_{2}=\frac{1-\phi_{1}\phi_{2}}{\tau_{1}\tau_{2}},$$

$$\mathcal{R}_{0}/N.$$

In this case, the disease recovery rate *γ* is identifiable as it was in the monophasic decay model. The combination *α*(*ηπ*_1_+(1−*η*)*π*_2_)*κρ* in the biphasic model has an analogous interpretation to that of the combination *απκρ* in the monophasic model. The two identifiable parameter combinations *ξ*_1_+*δ*_1_+*ξ*_2_+*δ*_2_ and (*ξ*_1_+*δ*_1_)(*ξ*_2_+*δ*_2_)−*δ*_1_*δ*_2_ come directly from the underlying biphasic pathogen decay model previously described in [18]; they are the sum and product of the apparent labile and persistent decay rates. These values characterize the observed pathogen decay, but they cannot attribute the values to the underlying processes, i.e., the same observed patterns could be generated by different values of the decay and phenotypic conversion parameters. Finally, because $\mathcal {R}_{0}/N$ is identifiable from case data, the basic reproduction number can be estimated if the population size is known.

Human disease surveillance provides us with information about five quantities, but, since there are eleven parameters, we can see that additional data will be needed to make inference about specific biological parameters, the persistence–infectivity trade-off, and the phenotype-specific risk. Quantitative microbial risk assessors often collect environmental samples to inform exposure estimates; such data could also be used to inform transmission models. Many quantification methods are either culture-based (which may not capture a dormant persistent phenotype) or PCR-based (which will not distinguish between phenotypes). If we combine case data with high-quality time-series environmental surveillance of the total pathogen population (*W*=*W*_1_+*W*_2_), such as might be collected to inform quantitative microbial risk assessment, we can additionally estimate

$$\alpha,$$

$$\frac{\tau_{1}(\eta+(1-\eta)\phi_{2})+\tau_{2}((1-\eta)+\eta \phi_{1})}{\tau_{1}\tau_{2}}.$$

By observing both case and environmental data, we can estimate the average shedding rate per volume *α*. This second parameter combination is less directly interpretable but could prove useful in estimating some biological parameters if others are known experimentally.

The underlying system dynamics are sufficiently complicated so that patterns of time-series case data and environmental surveillance, even though useful for characterizing the overall risk, do not fully reveal the biological processes or implications of the persistence–infectivity trade-off. However, if we have a way to estimate the relative abundance of the labile (*W*_1_) and persistent (*W*_2_) pathogen phenotypes in our environmental samples, we gain a great deal of parametric information. We can separately estimate *γ*,*α*,*η*,*δ*_1_,*δ*_2_,*ξ*_1_,*ξ*_2_,*κρπ*_1_, and *κρπ*_2_ (proof in supplementary material), at least in theory (there may be practical barriers for real-world data). With this information, we can infer the risk potentials of the labile and persistent phenotypes ($\mathcal {R}_{0,1}$ and $\mathcal {R}_{0,2}$).

These results suggest that there are two scientific strategies for understanding the underlying biological mechanisms and the persistence–infectivity trade-offs in this system. First, with high-quality case and environmental data that can distinguish between pathogen phenotypes, we can indirectly infer many of the biological parameter values. Second, if we cannot distinguish between pathogen phenotypes, usual disease environmental surveillance can be combined with targeted experimental studies designed to independently determine certain model parameters. Here, the important parameters to identify are the infectivity of pathogens in the persistent state (*π*_2_), the rates of entering dormancy (*δ*_1_) and of resuscitation (*δ*_2_), and the fraction of pathogens already dormant when initially shed into the environment (1−*η*)); identifying these parameters is essential to understanding the relative risks associated with the labile and persistent pathogen phenotypes. In particular, determining that one or more of these parameters is negligibly small provides a means to simplify the modeling framework. For example, if we can determine that resuscitation does not occur in the environment to an appreciable extent (*δ*_2_≈0), then the identifiable quantities from case data simplify to *γ*, *α*(*ηπ*_1_+(1−*η*)*π*_2_)*κρ*, *ξ*_1_+*δ*_1_,*ξ*_2_, and $\mathcal {R}_{0}/N$. The addition of environmental surveillance data helps to identify *α* and (after a little bit of algebra) *ηξ*_1_+(1−*η*)*ξ*_2_. Although determining that this one parameter *δ*_2_ is negligible would not fully resolve the persistence–infectivity question, it would simplify the remaining quantities and, consequently, future analysis. The specific experiments needed to estimate these parameters will likely vary by pathogen. Broadly speaking, however, animal challenge studies could be used to estimate *π*_1_ and *π*_2_, analysis of stool samples could be used to estimate *η*, and techniques designed to measure microbial dormancy could be harnessed to begin to better understand *δ*_1_ and *δ*_2_.

These scientific strategies are not mutually exclusive, and pursuing both population quantification and parameter determination strategies simultaneously will allow for corroboration and maximize our confidence in the conclusions of individual experimental studies because theoretical identifiability does not guarantee that real-world data will contain sufficient information to distinguish the mechanistic parameters in practice.

### The need for both disease surveillance and parameter data to elucidate mechanism: Simulation-based Shigella outbreak case study

In this section, we illustrate several of the theoretical results and demonstrate that disease incidence data can be used in conjunction with experimental studies to improve inference of model parameter values, e.g., by narrowing confidence intervals for estimates. Although the identifiable parameter combinations listed in the previous section represent the theoretical maximum amount of information that can be gleaned from observing certain system dynamics, in the real world with measurement error, the practical amount of information may be less. This point underscores the need for multiple approaches to corroborate estimates.

In this example, we simulate an outbreak of *Shigella* using the full biphasic decay disease model (Eq. (1)) for a small village with little-to-no drinking water treatment or sanitation infrastructure. In this scenario, villagers have unimproved sanitation or practice open defecation, allowing fecal matter and pathogens to enter the drinking water supply, which is a well-mixed lake adjacent to the village. Villagers do not treat their drinking water.

**Fig. 3:**
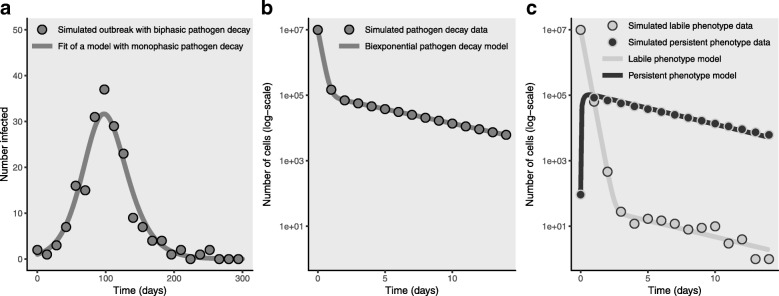
Pathogen decay dynamics cannot be inferred from case data alone. **a** An outbreak of *Shigella* simulated with the biphasic decay disease model with parameters *N*= 1000, *γ*= 1/6, *κ*= 8, *ρ*= 0.15, *V*= 4E8, *η*= 1-(1E-5), *π*_1_= 1.1E-2, *π*_2_= 1.1E-4, *ξ*_1_= 5, *ξ*_2_= 0.2, *δ*_1_= 0.05, *δ*_2_= 0.002, and $\mathcal {R}_{0}=$ 1.3. Biweekly case data were simulated from a binomial distribution. The monophasic decay disease model was fit to this simulated data using a binomial likelihood: parameter estimates are *ακρπ*= 3.4E-3 (95% CI: 2.8E-4, 4.1E-2), *γ*= 1.6E-1 (95% CI: 1.5E-1, 1.8E-1), *τ*= 6.3E-2 (95% CI: 5.2E-3, 7.6E-1), *I*(0)=1.0 (95% CI: 0.6,1.8). The asymptotic confidence intervals for *ακρπ* and *τ* are very wide. **b** Simulated pathogen decay data reveals a biphasic decay pattern and allows the estimation of the apparent fast and slow decay rates. The sum and product of these rates are represented by *ξ*_1_+*δ*_1_+*ξ*_2_+*δ*_2_= 5.24 and (*ξ*_1_+*δ*_1_)(*ξ*_2_+*δ*_2_)−*δ*_1_*δ*_2_=1.01. Using these estimates in fitting the biphasic decay disease model to the data allows us to make more accurate and precise inferences of certain parameter combinations: *ακρ*(*ηπ*_1_+(1−*η*)*π*_2_) (true: 1.09E-3, estimated: 1.18E-3 (95% CI: 1.02E-3, 1.36E-3)) and $\mathcal {R}_{0}/N$ (true: 1.30E-3, estimated: 1.40E-3 (95% CI: 1.27E-3, 1.53E-1)). **c** If we can separately quantify the labile and pathogen phenotypes in the pathogen decay experiment, we can estimate *ξ*_1_=4.96, *ξ*_2_=0.23, *δ*_1_=0.06, *δ*_2_=0.0018, all close to the true values. Using the biphasic decay disease model together with these estimates, we can estimate most of the remaining model parameters: *γ*=0.18, *κρα*=0.10, *η*=0.72, *π*_1_=1.11E-2, *π*_2_=6.66E-4. Only *η* is substantially different from it’s true value. With further shedding studies, we can estimate *η* and then characterize the labile disease risk ($\mathcal {R}_{0,1}$ estimated: 1.24, true: 1.30) and persistent disease risk ($\mathcal {R}_{0,1}$ estimated: 1.5E-2, true: 3.2E-3)

We consider the perspective of a researcher trying to use disease surveillance to elucidate the underlying dynamics of the outbreak. We fit the monophasic decay disease model (Eq. (1)) to the biweekly surveillance data (Fig. 3a). The monophasic environmentally mediated infectious disease transmission model captures the dynamics of the biphasic data well; i.e. we cannot detect the model misspecification from the fit alone. Indeed, fitting the biphasic decay model to this data negligibly improves the fit and predicts dynamics that are virtually indistinguishable from the monophasic model (not pictured). Even though we capture the dynamics with the monophasic model, our parameter estimates are highly uncertain. Asymptotic confidence intervals for the estimates of both *ακρπ* and *τ* span two orders of magnitude (Fig. 3 legend). In particular, the persistence *τ* can be arbitrarily small and still fit the data well.

One reasonable approach to improving our parameter inference here would be experimentally determining the environmental persistence of the pathogen. We conduct an experimental pathogen decay study, taking a sample of recently shed pathogen and observing its decay in a controlled environment (simulated in Fig. 3b). The pathogen decay study indicates that the pathogen decay is actually biphasic, which we did not detect from disease surveillance data alone. Fitting a biexponential model to this data allows us to estimate the sum and product of the apparent fast and slow decay rates (*ξ*_1_+*δ*_1_+*ξ*_2_+*δ*_2_) and (*ξ*_1_+*δ*_1_)(*ξ*_2_+*δ*_2_)−*δ*_1_*δ*_2_, respectively [18].

We can use this experimentally derived data in the parameter estimation for the biphasic decay disease model to circumvent the inference problems and estimate *γ*, *α*(*ηπ*_1_+(1−*η*)*π*_2_)*κρ*, and $\mathcal {R}_{0}/N$ with more accuracy and precision. The number of parameters (eleven), however, is greater than the number of degrees of freedom in the information (five), meaning that we can say very little about the values of the individual parameters, other than putting some general bounds on *τ*_1_,*τ*_2_, and *ακρ*.

Separate quantification of the labile and persistent phenotypes in the pathogen decay study (simulated in Fig. 3c), improves our understanding of the underlying dynamics substantially: we can estimate *ακρ*, *ξ*_1_, *ξ*_2_, *δ*_1_, *δ*_2_ with reasonable accuracy. However, this single pathogen decay study does not provide enough information to accurately estimate the fraction of pathogens shed into each phenotype *η*. By taking multiple measurements in a shedding study, we could estimate *η*, which would allow us to estimate (correctly) that the persistent phenotype accounts for less than 2% of the overall disease risk in this outbreak.

### Persistence–infectivity trade-offs affect outbreak dynamics

**Fig. 4:**
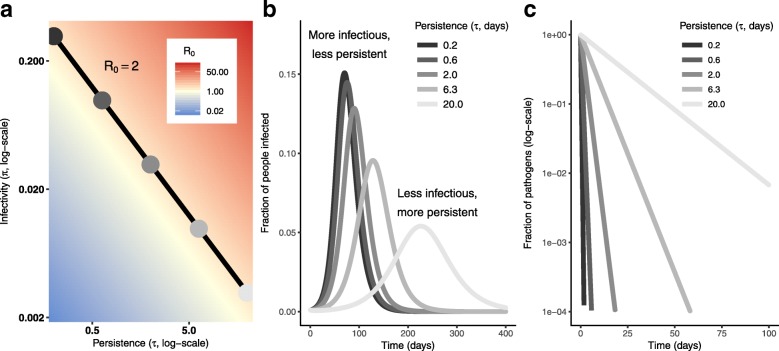
Infectivity–persistence trade-offs in a monophasic pathogen decay model. **a**. Heatmap of the basic reproduction number $\mathcal {R}_{0}$ of the monophasic decay disease model (Eq. (1)) as a function of persistence (*τ*) and infectivity (*π*). The line is the contour along which $\mathcal {R}_{0}=2$. Here, *N*= 1000, *γ*= 0.1, *κ*= 8, *ρ*= 0.15, *α*= 0.001. The colored dots correspond to the colored lines in (**b**) and (**c**). **b** Fraction of the population infected for the values of pathogen persistence (*τ*) and infectivity (*π*) given by the dots in (**a**). Although all points have $\mathcal {R}_{0}=2$, the epidemic dynamics vary significantly over the individual parameter values. **c** Pathogen decay curves in the absence of a system input illustrate the variation in the corresponding persistences (*τ*)

The pathogen infectivity *π* and the pathogen persistence *τ* are not part of the same identifiable parameter combinations in the monophasic decay model. This means that differences in the outbreak dynamics can be observed when we compare a highly infectious pathogen with low persistence to a less infectious pathogen with high persistence. At the same time, infectivity *π* and persistence *τ* appear in a product in the analytic equation for $\mathcal {R}_{0}$ (Eq. (2)). As long as the product of *π* and *τ* is constant, the basic reproduction number, and, therefore, the attack ratio, will be the same. Altogether, the persistence–infectivity trade-off can produce a variety of dynamics all associated with the same $\mathcal {R}_{0}$, and we find that slower outbreaks with smaller peak sizes are associated with less infectious, more persistent pathogens (Fig. 4).

**Fig. 5:**
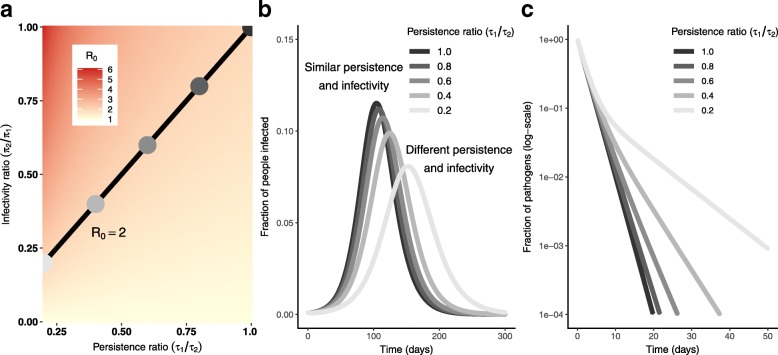
Infectivity–persistence trade-offs in a biphasic pathogen decay model. **a** Heatmap of the basic reproduction number $\mathcal {R}_{0}$ of the biphasic decay disease model (Eq. (1)) as a function of the ratio of the persistences (*τ*_1_/*τ*_2_) and infectivities (*π*_2_/*π*_1_). The line is the contour along which $\mathcal {R}_{0}=2$. The colored dots correspond to the colored lines in (**b**) and (**c**). **b** Fraction of the population infected over time for the values of pathogen persistence and infectivity ratios given by the dots in (**a**). Although all points have $\mathcal {R}_{0}=2$, the epidemic dynamics vary significantly over the parameter ratios. Here, *N*= 1000, *γ*= 0.1, *κ*= 8, *ρ*= 0.15, *α*= 0.001, *η*= 0.99, *ϕ*_1_= 0.1, *ϕ*_2_= 0.01, *π*_1_=0.0195, *τ*_1_=2. **c** Pathogen decay curves in the absence of a pathogen input illustrate the degree of biphasic behavrior corresponding to the persistence ratios (*τ*_1_/*τ*_2_)

In the biphasic decay disease model, we observe the same phenomena. Heuristically, the trade-offs are more easily observed if we express the degree of deviation from monophasic behavior using the ratios of infectivities *π*_2_/*π*_1_ and persistence times *τ*_1_/*τ*_2_, where more deviation from 1 indicates a greater deviation from monophasic behavior. Because we consider only the case where the persistent subpopulation is no less persistent and no more infectious than the labile subpopulation, we only consider 0<*π*_2_/*π*_1_<1 and 0<*τ*_1_/*τ*_2_<1. Rewriting the basic reproduction number in terms of these ratios,

7 $$ {\begin{aligned} \mathcal{R}_{0}&= \frac{\alpha\kappa\rho \pi_{1}\tau_{1} N}{\gamma}\left(\left(\frac{\eta+(1-\eta)\phi_{2}}{1-\phi_{1}\phi_{2}}\right) \right.\\& \left. + \frac{\pi_{2}/\pi_{1}}{\tau_{1}/\tau_{2}}\left(\frac{(1-\eta)+\eta \phi_{1}}{1-\phi_{1}\phi_{2}}\right)\right), \end{aligned}}  $$

we see that when we fix the persistence and infectivity of the labile subpopulation (*τ*_1_ and *π*_1_), the two ratios *π*_2_/*π*_1_ and *τ*_1_/*τ*_2_ must be proportional to maintain $\mathcal {R}_{0}$ (Fig. 5a). As with the monophasic model, the outbreak peaks later and smaller as the persistent pathogens become relatively more persistent but less infectious, (Fig. 5b). At the same time, the biphasic deviation become more pronounced (Fig. 5c).

Pathogens can fall in different places along the persistence–infectivity spectrum while still maintaining the same basic reproduction number, a proxy for pathogen fitness and measure of the attack ratio. Moreover, two phenotypes within a single population might likewise have different persistence–infectivity strategies, and thereby exhibit biphasic decay. In both the monophasic and biphasic decay disease models, these persistence–infectivity trade-offs have implications for the timing and peak size of the associated epidemics, which may in turn direct control strategies.

## Discussion

Microbial pathogens evolve or alter their metabolisms to maximize survival in response to stress. For pathogens that require a human host to reproduce, the resulting trade-offs will likely maximize transmission potential. These trade-offs can unfold over a long (evolutionary) time-scale causing differences to arise between populations or shorter (metabolic, gene expression, horizontal gene transfer [50], etc.) time-scales, allowing phenotypic variation to arise within a single population. We find that an epidemic-potential-preserving trade-off between persistence and infectivity affects the speed of epidemic dynamics. Highly infectious pathogens with low persistence have faster epidemic dynamics than persistent but less-infectious pathogens, even when the total risk is the same. Understanding the dynamics underlying a multiphenotype pathogen population is not possible with disease surveillance alone. Environmental surveillance of the total number of pathogens, with no distinction between subpopulations with different persistence phenotypes, is also not sufficient to uniquely estimate the underlying biological parameters. Selected experimental studies to ascertain key parameter values, on the other hand, can maximize the information available in disease and environmental surveillance and lead to fully specified risk models.

Understanding the data needs for a fully specified model will help inform risk assessments, which often do not consider heterogeneity of pathogen populations because of methodological and data limitations. Our analysis here demonstrates that characterizing the persistence–infectivity trade-off within pathogen populations in the environment can have important implications for risk assessment, particularly when biphasic decay is possible. Biphasic decay indicates the presence of labile and persistent phenotypes, each with a different associated disease potential (characterized by $\mathcal {R}_{0,1}$ and $\mathcal {R}_{0,2}$, respectively). Understanding how much the more persistent subpopulation contributes to the overall epidemic potential would improve the accuracy of risk assessments. Most risk assessments assume that pathogens decay exponentially (i.e., monophasically), discounting the possibility of a persistent subpopulation. Even when there is experimental evidence to characterize the persistences of the subpopulations, there has so far been little indication of whether to treat the persistent population as comparably infectious, not infectious, or somewhere in between. Experimental studies that provide information on the relative infectivity of the persistent subpopulation, as well as other biological parameters, will be useful for assessing the role of persistent subpopulations in public health risk assessment.

Questions of trade-offs between environmental persistence and infectivity are particularly relevant to the study of dormant microbial states—such as VBNC or antibiotic-resistant persister states—that result from environmental stresses. The VBNC state has been observed in many bacterial species and is characterized by a lack of culturability with classical techniques. Over fifty human pathogens have been reported to exhibit a VBNC state, including *E. coli*, *Salmonella*, and *Vibrio cholerae* [24, 26]. It is thought that the VBNC state is an adaptive strategy for survival in unfavorable environments, is induced by environmental stresses including disinfection, and can be reversed through resuscitation [24–26]. However, the exact role and mechanisms of the VBNC state appear to vary between species, so the VBNC state may be better considered a collection of related but species-specific states that are all characterized by a loss of culturability. Moreover, an emerging hypothesis, called the “dormancy continuum,” has suggested that the VBNC state and persister state are closely related phenomena [51, 52]. For most species, the pathogens in a dormant state have a slower die-off rate, and there is evidence of reduced infectivity in some species (e.g. *H. pylori* [31]). Additionally, there is evidence that cells can regain full virulence upon resuscitation [24–26], though in vivo resuscitation of ingested VBNC pathogens may be comparatively rare [53].

Fully describing the persistence–infectivity trade-offs and the underlying environmentally mediated pathogen dynamics require public health activities to go beyond collecting disease surveillance data and consider environmental processes. And, although the role of environmental surveillance will be central, experimentalists have the opportunity to expand not only our knowledge of the biology of human pathogens in the environment but also the implications for population-level disease by collaborating with mathematical disease modelers. Experimentalists can directly ascertain values of biological parameters, including the relative infectivity of persistent pathogens and the rates of phenotypic changes; calls for assessing rates of entering dormancy and resuscitation have already been made by mathematical modelers assessing persister cells [22]. Alternatively, by developing methods that separately quantify the dynamics of labile and persistent pathogens, experimentalists can help provide the time-series data modelers need to indirectly infer the biological parameters and rates. These direct and indirect pathways are complementary, and both should be pursued.

More generally, microbiologists could contribute to public health by focusing on the downstream implications of gene expression and metabolic processes. For example, how does the presence of a certain gene in a pathogen population translate into its ability to infect those exposed to it? Or, under which real-world environmental conditions should we expect extended persistence of still-infectious pathogens? Shifting the experimental mindset to answering such questions will significantly benefit risk assessment and public health researchers.

The combination of human and environmental surveillance data, experimental data, and mechanistic models through a dialectic process promises to move theory and application towards more informed public health decision making. At the same time we always need to be aware of the assumptions behind a specific mechanistic model structure. For example, we used the basic reproduction number $\mathcal {R}_{0}$ as a proxy for pathogen fitness. Although $\mathcal {R}_{0}$ is likely an acceptable first approximation for pathogen fitness, real-world trade-offs may not precisely maintain the basic reproduction number because of the complex nature of genetic or metabolic trade-offs and because bacteria are not inherently optimizing the abstract concept of human disease transmission (there may be local, contextual effects or the influence of other hosts). Also, by using a compartmental model, we make implicit assumptions about deterministic and well-mixed dynamics. True dynamics are likely to be spatial (perhaps related to biofilm formation) and likely stochastic; indeed, persister cells, for example, are thought to arise from stochastic fluctuations in gene expression and to comprise only a small fraction of the population, on the order of 10^-4^ to 10^-6^. Nevertheless, our work provides a first mathematical modeling framework for considering possible population-level public health implications of microbial dormancy.

## Conclusion

To improve microbial risk assessments of environmentally mediated pathogens and to provide a more precise means of developing environmentally-based control strategies, we will require a better understanding of the dynamic mechanisms that drive the variation in pathogen persistence times, as well as the associated trade-offs with infectivity. The development and analysis of the dynamic models presented here create a framework to translate data obtained from microbial ecological and experimental research into predictions of population-level public health outcomes. In particular, targeted studies designed to elucidate the dynamics of persistent pathogen subpopulations are needed.

## Additional files

Additional file 1 Supplementary material. This document provides proofs of theoretical results, including the calculation of the basic reproduction number and the identifiability analysis. (PDF 134 kb)

Additional file 2 Additional proof 1. This file is a Mathematica notebook providing computations for a proof described in Additional file 1. Here, we consider data of the form *I*. (NB 141 kb)

Additional file 3 Additional proof 2. This file is a Mathematica notebook providing computations for a proof described in Additional file 1. Here, we consider data of the form *I* and *W*. (NB 131 kb)

Additional file 4 Additional proof 3. This file is a Mathematica notebook providing computations for a proof described in Additional file 1. Here, we consider data of the form *I* and *W*_1_. (NB 161 kb)

Additional file 5 Additional proof 4. This file is a Mathematica notebook providing computations for a proof described in Additional file 1. Here, we consider data of the form *I*, *W*_1_, and *W*_2_. (NB 39 kb)
