# Supplementary material for: Phenotypic variations in persistence and infectivity between and within environmentally transmitted pathogen populations impact population-level epidemic dynamics
Source: BMC Infect Dis. 2019 May 22;19:449. doi: 10.1186/s12879-019-4054-8 (PMC6530054; doi:10.1186/s12879-019-4054-8)
Supplement: Supplementary file 1 — Supplementary material. This document provides proofs of theoretical results, including the calculation of the basic reproduction number and the identifiability analysis. (PDF 134 kb) [file 12879_2019_4054_MOESM1_ESM.pdf]

Phenotypic variations in persistence and infectivity between and within environmentally transmitted pathogen populations impact population-level epidemic dynamics: Supplementary material

1. Basic reproduction number of the biphasic decay disease model

Proposition 1. *The basic reproduction number for the environmentally mediated infectious disease transmission model with biphasic pathogen decay (Eqs. 3) is*

$$\mathcal{R}_0 = \frac{\alpha\kappa\rho N}{\gamma}\left(\pi_1\tau_1\left(\frac{\eta+(1-\eta)\phi_2}{1-\phi_1\phi_2}\right)+\pi_2\tau_2\left(\frac{(1-\eta)+\eta\phi_1}{1-\phi_1\phi_2}\right)\right).$$

Proof. We compute the basic reproduction number of the biphasic decay disease model using the Next Generation Method. The equations of the biphasic decay disease model are

$$\begin{aligned}
\dot{S} &= -\kappa\rho S(\pi_1 W_1+\pi_2 W_2),\\
\dot{I} &= \kappa\rho S(\pi_1 W_1+\pi_2 W_2)-\gamma I,\\
\dot{R} &= \gamma I,\\
\dot{W}_1 &= \alpha\eta I+\delta_2 W_2-(\xi_1+\delta_1)W_1,\\
\dot{W}_2 &= \alpha(1-\eta)I+\delta_1 W_1-(\xi_2+\delta_2)W_2.
\end{aligned} \tag{S1}$$

Denote $x=(I,W_1,W_2)$ be the disease compartments and $y=(S,R)$ the non-disease compartments. Then, we may write

$$\dot{x}=\mathcal{F}-\mathcal{V} \tag{S2}$$

where

$$\mathcal{F}=\begin{bmatrix}\kappa\rho S(\pi_1 W_1+\pi_2 W_2)\\ 0\\ 0\end{bmatrix}, \tag{S3}$$

$$\mathcal{V}=\begin{bmatrix}\gamma I\\ -\alpha\eta I-\delta_2 W_2+(\xi_1+\delta_1)W_1\\ -\alpha(1-\eta)I-\delta_1 W_1+(\xi_2+\delta_2)W_2\end{bmatrix}. \tag{S4}$$

Then, we have new-infection (F) and compartment transfer (V) matrices

$$F = \begin{bmatrix} 0 & \kappa\rho N\pi_1 & \kappa\rho N\pi_2 \\ 0 & 0 & 0 \\ 0 & 0 & 0 \end{bmatrix}, \tag{S5}$$

$$V = \begin{bmatrix} \gamma & 0 & 0 \\ -\alpha\eta & \xi_1 + \delta_1 & -\delta_2 \\ -\alpha(1-\eta) & -\delta_1 & \xi_2 + \delta_2 \end{bmatrix}. \tag{S6}$$

Then,

$$V^{-1} = \begin{bmatrix} \frac{1}{\gamma} & 0 & 0 \\ \frac{\alpha\eta(\xi_2+\delta_2)+\alpha(1-\eta)\delta_2}{\gamma((\xi_1+\delta_1)(\xi_2+\delta_2)-\delta_1\delta_2)} & \frac{\xi_2+\delta_2}{(\xi_1+\delta_1)(\xi_2+\delta_2)-\delta_1\delta_2} & \frac{\delta_2}{(\xi_1+\delta_1)(\xi_2+\delta_2)-\delta_1\delta_2} \\ \frac{\alpha_(1-\eta)(\xi_1+\delta_1)+\alpha\eta\delta_1}{\gamma((\xi_1+\delta_1)(\xi_2+\delta_2)-\delta_1\delta_2)} & \frac{\delta_1}{(\xi_1+\delta_1)(\xi_2+\delta_2)-\delta_1\delta_2} & \frac{\xi_1+\delta_1}{(\xi_1+\delta_1)(\xi_2+\delta_2)-\delta_1\delta_2} \end{bmatrix}. \tag{S7}$$

Substituting

$$\tau_1 = \frac{1}{\xi_1 + \delta_1} \tag{S8}$$

$$\tau_2 = \frac{1}{\xi_2 + \delta_2}, \tag{S9}$$

$$\phi_1 = \frac{\delta_1}{\xi_1 + \delta_1}, \tag{S10}$$

$$\phi_2 = \frac{\delta_2}{\xi_2 + \delta_2}, \tag{S11}$$

we write

$$V^{-1} = \begin{bmatrix} \frac{1}{\gamma} & 0 & 0 \\ \frac{\alpha(\eta+(1-\eta)\phi_2)\tau_1}{\gamma(1-\phi_2\phi_2)} & \frac{\tau_1}{1-\phi_2\phi_2} & \frac{\phi_2\tau_1}{1-\phi_2\phi_2} \\ \frac{\alpha((1-\eta)+\eta\phi_1)\tau_2}{\gamma(1-\phi_2\phi_2)} & \frac{\phi_1\tau_2}{1-\phi_2\phi_2} & \frac{\tau_2}{1-\phi_2\phi_2} \end{bmatrix}. \tag{S12}$$

Then, the next generation matrix is $K = FV^{-1}$,

$$K = \begin{bmatrix} \frac{\alpha\kappa\rho N}{\gamma}\left(\frac{(\eta+(1-\eta)\phi_2)\pi_1\tau_1+((1-\eta)+\eta\phi_1)\pi_2\tau_2}{1-\phi_2\phi_2}\right) & \kappa\rho N\left(\frac{\pi_1\tau_1+\pi_2\phi_1\tau_2}{1-\phi_2\phi_2}\right) & \kappa\rho N\left(\frac{\pi_1\phi_2\tau_1+\pi_2\tau_2}{1-\phi_2\phi_2}\right) \\ 0 & 0 & 0 \\ 0 & 0 & 0 \end{bmatrix}, \tag{S13}$$

and $\mathcal{R}_0$ is the spectral radius of K, namely

$$\mathcal{R}_0 = \frac{\alpha\kappa\rho N}{\gamma}\left(\pi_1\tau_1\left(\frac{\eta + (1-\eta)\phi_2}{1-\phi_1\phi_2}\right) + \pi_2\tau_2\left(\frac{(1-\eta)+\eta\phi_1}{1-\phi_1\phi_2}\right)\right). \tag{S14}$$

□

2. Identifiability analysis

We use a differential algebra approach to identifiability to find an input–output equation, which is a monic, polynomial equation that can be written in terms of only the data variable (and its derivatives) and that has equivalent observed dynamics to that of the original ODE system. The coefficients of the input–output equation are the identifiable parameter combinations.

We illustrate the method for the monophasic decay model in the following proposition, which is adapted from Eisenberg et al. [S1]. The proof is adapted from the supplementary material of Brouwer et al. [S2]

Proposition. *The identifiable combinations of the model given in (Eqs (1)) given time series data of prevalence of infected individuals I are $\alpha\pi\kappa\rho$, τ, and γ. If the time series of the environmental compartment W is also observed, then α is separately identifiable.*

Proof. First, we prove the theorem for prevalence data I. The model equations are

$$0 = \dot{S} + \kappa\rho\pi SW \tag{S15}$$
$$0 = \dot{I} - \kappa\rho\pi SW + \gamma I \tag{S16}$$
$$0 = \dot{R} - \gamma I \tag{S17}$$
$$0 = \dot{W} - \alpha I + \xi W \tag{S18}$$

Eq. (S17) gives us no parametric information. Solving Eq. (S16) for S, $S = \frac{\dot{I}+\gamma I}{\pi\kappa\rho W}$, the other two equations simplify to

$$0 = \ddot{I}W - \kappa\rho\pi IW^2 + (\xi+\gamma)\dot{I}W - \alpha\dot{I}I + \gamma\kappa\rho\pi IW^2 + \gamma\xi IW - \alpha\gamma I^2 \tag{S19}$$
$$0 = \dot{W} - \alpha I + \xi W \tag{S18}$$

If both I and W are observed, these two equations are input–ouput equations, and we can read the identifiable parameter combinations from the coefficients, namely γ, $\tau = 1/\xi$, α, and $\kappa\rho\pi$. However, if W is not observed, we must further reduce the system.

Using Ritt's pseudodivision [S3] (steps provided in accompanying Mathematica notebook, "Monophasic_model_identifiability"), we arrive at the following input–output equation:

$$
\begin{aligned}
0 &= 4\alpha^2\gamma^3\kappa\rho^2\pi^2 I^6 + 12\alpha^2\gamma^2\kappa\rho^2\pi^2 I^5\dot{I} + \alpha\gamma^3\kappa\rho\xi^2\pi I^5 + 12\alpha^2\gamma\kappa\rho^2\pi^2 I^4\dot{I}^2 \\
&+ \left(2\alpha\kappa\rho\xi\pi\gamma^3 + 3\alpha\kappa\rho\xi^2\pi\gamma^2\right) I^4\dot{I} + \left(4\alpha\kappa\rho\pi\gamma^3 + 6\alpha\kappa\rho\xi\pi\gamma^2\right) I^4\ddot{I} \\
&+ 4\alpha\gamma^2\kappa\rho\pi I^4 I^{(3)} + 4\alpha^2\kappa\rho^2\pi^2 I^3\dot{I}^3 + \left(3\alpha\gamma\kappa\rho\xi^2\pi - 4\alpha\gamma^3\kappa\rho\pi\right) I^3\dot{I}^2 \\
&+ \left(2\alpha\kappa\rho\pi\gamma^2 + 12\alpha\kappa\rho\xi\pi\gamma\right) I^3\dot{I}\ddot{I} + 8\alpha\gamma\kappa\rho\pi I^3\dot{I}I^{(3)} - 3\alpha\gamma\kappa\rho\pi I^3\ddot{I}^2 \\
&+ \left(\xi^2\gamma^3 + \xi^3\gamma^2\right) I^3\ddot{I} + \gamma^2\xi^2 I^3 I^{(3)} - \left(6\alpha\kappa\rho\pi\gamma^2 + 6\alpha\kappa\rho\xi\pi\gamma - \alpha\kappa\rho\xi^2\pi\right) I^2\dot{I}^3 \\
&+ (6\alpha\kappa\rho\xi\pi - 2\alpha\gamma\kappa\rho\pi) I^2\dot{I}^2\ddot{I} + 4\alpha\kappa\rho\pi I^2\dot{I}^2 I^{(3)} - \left(\xi^3\gamma^2 + \gamma^3\xi^2\right) I^2\dot{I}^2 - 3\alpha\kappa\rho\pi I^2\dot{I}\ddot{I}^2 \\
&+ \left(\xi\gamma^3 + 2\xi^2\gamma^2 + 2\xi^3\gamma\right) I^2\dot{I}\ddot{I} + \left(\xi\gamma^2 + 2\xi^2\gamma\right) I^2\dot{I}I^{(3)} + \left(\gamma^3 + 2\xi\gamma^2 + \xi^2\gamma\right) I^2\ddot{I}^2 \\
&+ \left(2\gamma^2 + 2\xi\gamma\right) I^2\ddot{I}I^{(3)} + \gamma I^2 (I^{(3)})^2 - (3\alpha\gamma\kappa\rho\pi + 4\alpha\kappa\rho\xi\pi) I\dot{I}^4 \\
&- \left(3\xi^2\gamma^2 + 2\xi^3\gamma + \gamma^3\xi\right) I\dot{I}^3 - \left(2\gamma^3 + 3\xi\gamma^2 + \xi^2\gamma - \xi^3\right) I\dot{I}^2\ddot{I} + \left(\xi^2 - 2\gamma^2\right) I\dot{I}^2 I^{(3)} \\
&- \left(2\gamma^2 + \xi\gamma - \xi^2\right) I\dot{I}\ddot{I}^2 + (2\xi - \gamma) I\dot{I}\ddot{I}I^{(3)} + I\dot{I}(I^{(3)})^2 - (\gamma + \xi) I\ddot{I}^3 - I\ddot{I}^2 I^{(3)} - \alpha\kappa\rho\pi\dot{I}^5 \\
&+ \left(\gamma^3 - 2\xi^2\gamma - \xi^3\right) \dot{I}^4 + \left(2\gamma^2 - \xi\gamma - 2\xi^2\right) \dot{I}^3\ddot{I} - (\gamma + \xi)\dot{I}^3 I^{(3)} + 2\gamma\dot{I}^2\ddot{I}^2 - \dot{I}^2\ddot{I}I^{(3)} + \dot{I}\ddot{I}^3
\end{aligned}
\tag{S20}
$$

Because this equation is an input–output equation (i.e. a monic polynomial in the data I and its derivatives that has observable dynamics equivalent to the original model), the coefficients are identifiable. That is $\alpha\kappa\rho\pi$, $\tau = 1/\xi$, and γ are structurally identifiable when I is observed. □

Proposition 2. *The structurally identifiable parameter combinations for the environmentally mediated infectious disease transmission model with biphasic pathogen decay (Eqs. 2) if case data (I) are observed are*

$$
\begin{gathered}
\gamma, \\
\alpha(\eta\pi_1 + (1-\eta)\pi_2)\kappa\rho, \\
\xi_1 + \delta_1 + \xi_2 + \delta_2 = \frac{\tau_1 + \tau_2}{\tau_1\tau_2}, \\
(\xi_1 + \delta_1)(\xi_2 + \delta_2) - \delta_1\delta_2 = \frac{1 - \phi_1\phi_2}{\tau_1\tau_2}, \\
\mathcal{R}_0/N.
\end{gathered}
$$

If the total pathogen concentration is also observed ($W = W_1 + W_2$), then

$$
\begin{gathered}
\alpha, \\
\frac{\tau_1(\eta + (1-\eta)\phi_2) + \tau_2((1-\eta) + \eta\phi_1)}{\tau_1\tau_2},
\end{gathered}
$$

are also structurally identifiable. If the persistent pathogens are not culturable by usual methods and only labile pathogen concentration (W_1) is observed, then the additional identifiable parameter combinations are instead

$$\alpha\eta,$$
$$\frac{1}{\eta}\frac{\tau_1(\eta + (1-\eta)\phi_2)}{\tau_1\tau_2}.$$

One caveat to this last result is that we cannot distinguish W from W_1 using case and environmental monitoring data alone. If the labile (W_1) and persistent (W_2) pathogens can be separately quantified, then γ, α, η, δ_1, δ_2, ξ_1, ξ_2, $\kappa\rho\pi_1$, and $\kappa\rho\pi_2$ are structurally identifiable.

Mathematica notebooks proving Proposition 2 are provided in accompanying Mathematica notebooks

- "Biphasic_model_identifiability_data_I",
- "Biphasic_model_identifiability_data_IW",
- "Biphasic_model_identifiability_data_IW1", and
- "Biphasic_model_identifiability_data_IW1W2."

References

[S1] Eisenberg MC, Robertson SL, Tien JH. Identifiability and estimation of multiple transmission pathways in cholera and waterborne disease. Journal of Theoretical Biology. 2013;324:84–102.

[S2] Brouwer AF, Weir MH, Eisenberg MC, Meza R, Eisenberg JNS. Dose-response relationships for environmentally mediated infectious disease transmission models. PLOS Computational Biology. 2017;13(4):e1005481.

[S3] Meshkat N, Anderson C, DiStefano JJ. Alternative to Ritt's pseudodivision for finding the input-output equations of multi-output models. Mathematical Biosciences. 2012;239(1):117–123.
